# Subcellular Localization of *Arabidopsis* Pathogenesis-Related 1 (PR1) Protein

**DOI:** 10.3390/ijms18040825

**Published:** 2017-04-13

**Authors:** Tamara Pečenková, Roman Pleskot, Viktor Žárský

**Affiliations:** 1 Laboratory of Cell Biology, Institute of Experimental Botany, Academy of Sciences of the Czech Republic, Rozvojova 263, 165 02 Prague 6, Czech Republic; pleskot@ueb.cas.cz; 2 Laboratory of Cell Morphogenesis, Department of Experimental Plant Biology, Faculty of Science, Charles University in Prague, Vinicna 5, 128 44 Prague 2, Czech Republic; zarsky@ueb.cas.cz

**Keywords:** PR1, MVB, PI(3)P, CAPE, secretion, trafficking

## Abstract

The *Arabidopsis* *thaliana* pathogenesis-related 1 (PR1) is an important defense protein, so far it has only been detected in extracellular space and its subcellular sorting and transport remain unexplained. Using a green fluorescent protein (GFP) tagged full length, as well as a C-terminus truncated version of PR1, we observed that when expressed ectopically in *Nicotiana benthamiana* leaves, PR1 co-localizes only partially with Golgi markers, and much more prominently with the late endosome (LE)/multivesicular body (MVB) FYVE marker. The C-truncated version PR1ΔC predominantly localized to the endoplasmic reticulum (ER). The same localizations were found for stable *Arabidopsis* transformants with expression of PR1 and PR1ΔC driven by the native promoter. We conclude that the *A. thaliana* PR1 (AtPR1) undergoes an unconventional secretion pathway, starting from the C-terminus-dependent sorting from the ER, and utilizing further transportation via phosphatidyl-inositol-3-phosphate (PI(3)P) positive LE/MVB-like vesicles. The homology model of the PR1 structure shows that the cluster of positively charged amino acid residues (arginines 60, 67, 137, and lysine 135) could indeed interact with negatively charged phospholipids of cellular membranes. It remains to be resolved whether Golgi and LE/MVB localization reflects an alternative sorting or trafficking succession, and what the role of lipid interactions in it will be.

## 1. Introduction

In plants, the synthesis of various pathogenesis-related (PR) proteins is among the first of the biotic stress-induced responses. There are 17 evolutionarily conserved families of PR proteins. Some of them have direct antimicrobial function while others play a role as immunity signaling molecules [1,2]. 

PR1s are the most abundantly produced PR proteins upon pathogen attack (e.g., 2%–10% of the total tobacco leaf protein; [3,4]). Members of the PR1 family are highly conserved among plants; homologues have also been found in fungi, insects, and vertebrates including human. Together they form a superfamily of secreted proteins named CAP (from cystein rich secretory protein (CRISP), antigen 5, and PR1 proteins; [5]). Even though PR1s have been extensively studied, their exact function remains enigmatic. They have been suggested to have antifungal activity, or to play a role in host salicylic acid (SA)-mediated defense signaling and hypersensitive response (HR)-related cell death [6]. 

PR1s are present in plant genomes as highly multiplied gene families. In the genome of *Arabidopsis thaliana*, there are 22 PR1-type genes [7], mostly arranged in clusters of genes [6]. Only one of them, *A. thaliana* PR1 (AtPR1, At2g14610), is activated by pathogens, insects, or chemical treatments, whereas other PR1-type genes are constitutively expressed in roots and pollen [6]. 

There is very little data on the cell biology of PR1s in plants. In *Arabidopsis*, PR1 protein tagged with mCherry was found to accumulate in high levels in the apoplastic space of the epidermis of cotyledons of wild-type seedlings, with only a weak signal detected in the vacuolar lumen [8]. However, in the mutant of KEEP ON GOING (KEG), a RING E3 ligase involved in abscisic acid signaling during growth and development [9], the vacuolar PR1-mCherry signal appeared much stronger. 

A member of another class of PR proteins, defensin protein PDF1.2, tagged with green fluoresecent protein (GFP) and overexpressed in *Arabidopsis*, localizes in the endoplasmic reticulum (ER)-derived structures called ER bodies [10,11,12]. However, after a fungal attack in the presence of glucose which induces a specific invasion strategy of *Colletotrichum gloeosporioides*, the PDF protein is secreted into the apoplastic space [12]. This unconventional mode of secretion was named enhanced secretion of proteins localized in ER bodies (ESPER), and was also found to be employed in protein PR1 secretion. ESPER was not commonly induced by inoculation of tested nonadapted *Colletotrichum* species, nor with flg22. The presence of a signal peptide was insufficient for sorting to ESPER. This suggests that some features of PDF1.2a and PR1 in their amino acid or glycosylation patterns are important for ESPER [12].

The data coming from other plant models deal more with PR1 proteins possible function in defense. When PR1 genes were over-expressed in tobacco, the transformed plants were slightly more resistant to certain pathogens [13] and conversely, plants with silenced PR1s were more susceptible than wild-type, e.g., to the oomycete *Phytophthora parasitica*, but displayed an unaffected systemic acquired resistance (SAR) [14]. Silencing also resulted in an increase of apoplastic *b*-(1/3)-glucanase activity and a decrease in callose deposition [14]. 

Recently, an 11-amino acid C-terminal CAP-derived peptide 1-like (CAPE1-like) portion of the tomato preproprotein PR1b containing the characteristic PxGNxxxxxPY motif, was discovered to function as a defense signaling peptide [15]. Tomato plants presprayed with CAPE1 exhibited increased resistance to the bacterial pathogen *Pseudomonas syringae* pv. *tomato* DC3000 and reduced *Spodoptera litura* larval growth and weight. It was suggested that this peptide probably functions as a novel DAMP (damage-associated molecular pattern) inducer of immunity. AtCAPE1 has been also found to function as a negative regulator of salt stress [16].

In our work, using the C-terminal fusion of *Arabidopsis* PR1 protein At2g14610 with a GFP tag, we tried to detect which compartments along the secretory pathway are employed by cells in order to secret PR1 into apoplast. We have tested our construct both by transient expression in *Nicotiana benthamiana* and by stable expression in *A. thaliana* under 35S as well as an endogenous promoter. We have observed that in transient ectopic expression in *N. benthamiana* leaves, PR1 is transported via an unconventional pathway as it co-localizes only partially with Golgi markers, and much more prominent with the late endosome (LE)/multivesicular body (MVB) FYVE marker. Expression in *Arabidopsis* under the native promoter and overexpression under the 35S promoter in *Arabidopsis* confirmed compartmental localization found in transient expression. We conclude that the AtPR1 ER sorting is dependent on the motif localized on C-terminus and that major part of PR1 in the cell might be secreted via an unconventional route of secretion that transits phosphatidyl-inositol-3-phosphate (PI(3)P) positive LE/MVB vesicles.

## 2. Results

### 2.1. Localization of AtPR1 after the Transient Expression in N. benthamiana

**Figure 1:**
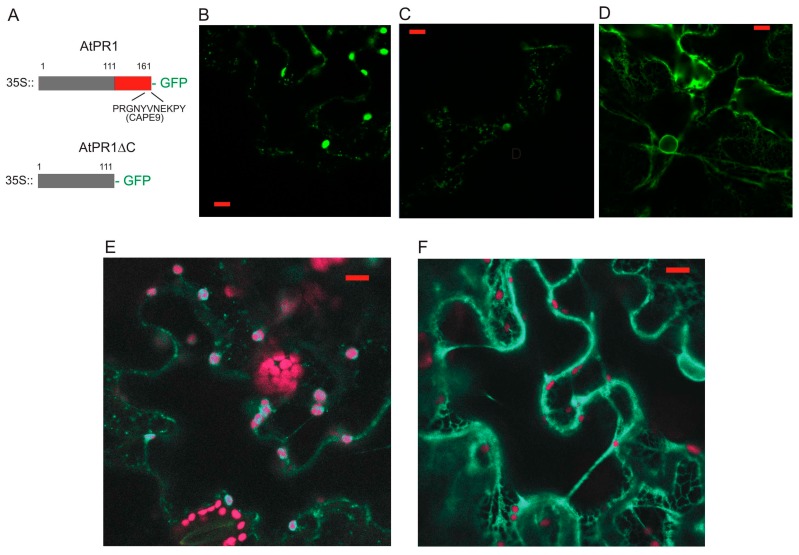
(**A**) A schematic view of pathogenesis-related 1 (PR1) protein and its C-deleted version (PR1ΔC). Numbers of the first and last amino acid positions are shown, as well as the full sequence of C-terminal CAPE9 peptide (homolog of CAPE1). (**B**,**C**) PR1 protein fused to green fluorescent protein (GFP) transiently expressed in *Nicotiana benthamiana*, (**C**) shows abundant vesicle-like signals in the cortical cytoplasm. (**D**) PR1ΔC-GFP localizes to endoplasmic reticulum (ER)-like structure. (**E**) Lambda scan showing the difference between the punctate PR1-GFP signal (green) and autofluorescence of plastids (red). (**F**) Lambda scan confirming that the observed signal on ER comes from the PR1ΔC-GFP. Scale bar: 10 μm.

As the AtPR1 protein fused at the C-terminus to GFP was transiently expressed in *N. benthamiana*, we observed the GFP signal in compartments localized throughout the cytoplasm (Figure 1A–C). We have also designed a truncated version of PR1 with deleted 50 amino acids from the C-terminus and fused it in frame again C-terminally to GFP (AtPR1ΔC; Figure 1A,D,F). We expected that this protein will be predominantly secreted outside the cell, since it has no other known sorting signal. Surprisingly, the majority of the protein seemed to be retained in the ER and AtPR1ΔC-GFP positive compartments are not observed in contrast to AtPR1-GFP. This is supported by the observation of a strong nuclear membrane signal in AtPR1ΔC-GFP possibly coming from an over-loaded ER (Figure 1D). In order to confirm the presence of the GFP signal, we have checked the emission spectra by a lambda scan mode (Figure 1E,F), allowing us to distinguish between the real GFP signal and chloroplast autofluorescence.

The PR1-positive compartments were found to be highly mobile and of variable shape, with a diameter of approximately a few hundred nanometer up to one micrometer big (Video S1). Video S1 also indicates a possible occurrence of dilated subdomains of ER in transiently expressing *N. benthamiana* cells. As PR1 was previously reported to be localized extracellularly [8,12], in order to better visualize cell wall GFP signal, we subjected leaves to increased pH, as well as to plasmolysis by a high concentration of mannitol or NaCl. We observed a weak cell wall and a plasmamembrane signal after the mannitol induced plasmolysis, for both full length and C-truncated forms of PR1 (Figure S1). 

Based on these observations, we concluded that the AtPR1 protein is localized to a vesicle-like intracellular compartments and that localization into these compartments requires an intact C-terminus in transient *N. benthamiana* cell expression. PR1-GFP is also localized adjacent to the plasma membrane and weakly into the apoplastic cell wall space. 

### 2.2. Co-Localization of PR1 with Compartmental Markers in N. benthamiana Transiently Transformed Cells

**Figure 2:**
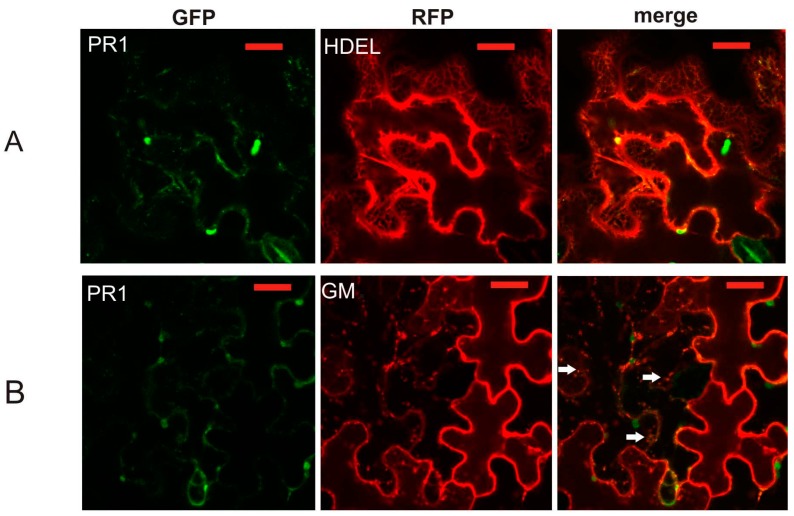

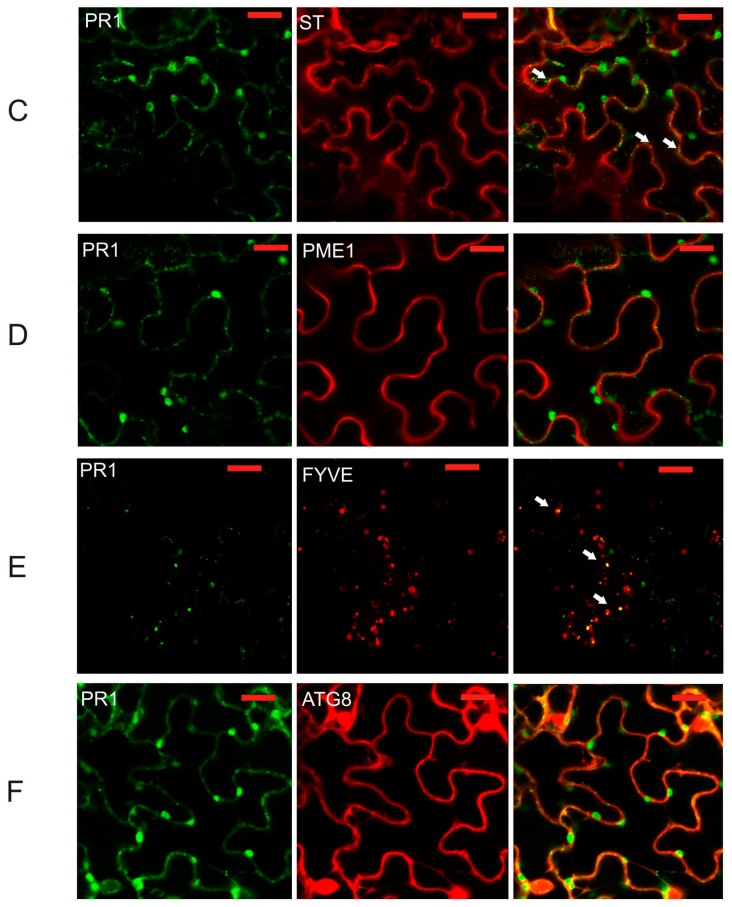
Co-localization of PR1-GFP with compartmental markers. (**A**) ER marker fluorophore dsRed with ER retention signal HDEL (dsRed-HDEL); (**B**) *cis*-Golgi marker Golgi mannosidase GM-RFP; (**C**) *trans*-Golgi marker sialyl transferase-RFP (ST-RFP); (**D**) extracellular marker pectin methyl esterase 1-RFP (PME1-RFP); (**E**) phosphatidyl-inositol-3-phosphate(PI(3)P)-enriched early/late endosomal compartmental marker dsRed-FYVE and (**F**) autophagy marker ATG8-RFP. For **B**, **C** and **E** arrows point to co-localization events. Scale bar: 20 μm.

**Figure 3:**
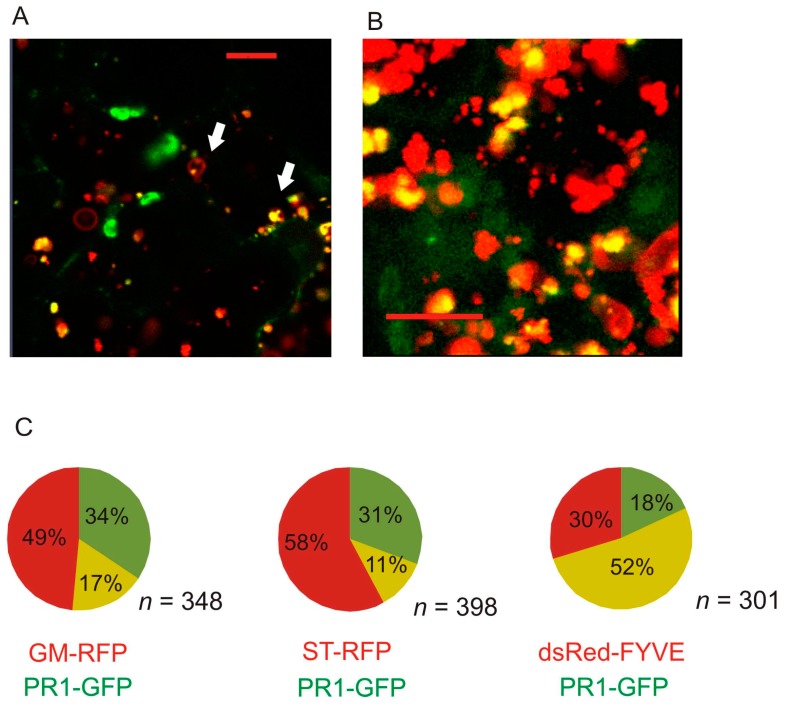
(**A**) The signal overlap for *Arabidopsis thaliana* PR1 (AtPR1) and FYVE-positive compartment. Arrows point to compartments with a multilayer structure described for multivesicular bodies, with obvious presence of particles/vesicles within the vesicle. (**B**) The three-dimensional (3D) reconstruction of Z-stack scanning of these compartments. Note that the GFP signal is surrounded by a red signal of FYVE. The shape of the vesicles is irregular partially because of their rapid movement which disturbed more precise 3D reconstruction. Scale bar: 10 μm. (**C**) Quantification of colocalization of PR1-positive compartment overlap with *cis*- and *trans*-Golgi markers (GM and ST, respectively), as well as with FYVE. Red sector: Proportion of RFP/dsRed signal; Green sector: Proportion of GFP signal; Yellow sector: Proportion of co-localizing RFP/dsRed and GFP signals out of the total number of analyzed objects (*n*). The quantification was performed on at least 10 regions (50 × 50 μm) out of five different scans for each of the three co-localization analyses.

In order to uncover the identity of the AtPR1-positive compartment, we have performed co-localization studies of full-length PR1 using red fluorescent protein (RFP)-tagged markers for ER (dsRed-HDEL; [17]), *cis*-Golgi marker Glycine max Golgi mannosidase-RFP (GM-RFP; [18]), *trans*-Golgi marker sialyl transferase-RFP (ST-RFP; donated by Ian Moore, Oxford), extracellular marker pectin methyl esterase 1–RFP (PME1-RFP; [19]), autophagy marker ATG8-RFP [20], and PI(3)P–enriched early/late endosomal compartmental marker dsRed-FYVE [21] (Figure 2A–F). PR1-GFP only weakly colocalized with the ER (Figure 2). The overlap with the Golgi marker was partial and the majority of the signal overlapped with FYVE-positive compartment (Figure 2). In some cases, these compartments had obvious structure, typical for MVBs—the presence of particles/vesicles within the vesicle (Figure 3). Unfortunately, our time series scanning did not reveal any events resembling exosome secretion, fusion with vacuolar membrane, nor endocytotic events (Video S2). We performed the object-based quantification of colocalization of PR1-GFP with GM-RFP, ST-RFP and dsRed-FYVE. The highest proportion of the green signal overlapping with the red one was found for the combination of PR1-GFP with the dsRed-FYVE (Figure 3). The PR1 obviously does not share the secretion route with PME1 (Figure 2D). No distinct co-localization with the ATG8 autophagy marker was observed (Figure 2F). Even though transiently expressed proteins are found in cells affected by pathogen attack (*Agrobacterium*) and have increased expression of stress-related PR1 protein, this situation does not seem to lead to a distinct increase of autophagy. In the cells observed, the ATG8 signal is mostly cytoplasmic with only a few ATG8-RFP positive autophagosomal bodies that had only partial co-localization with the PR1-GFP-positive vesicles (Figure 2F). 

**Figure 4:**
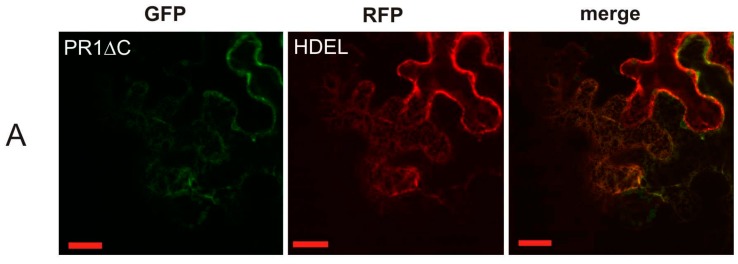

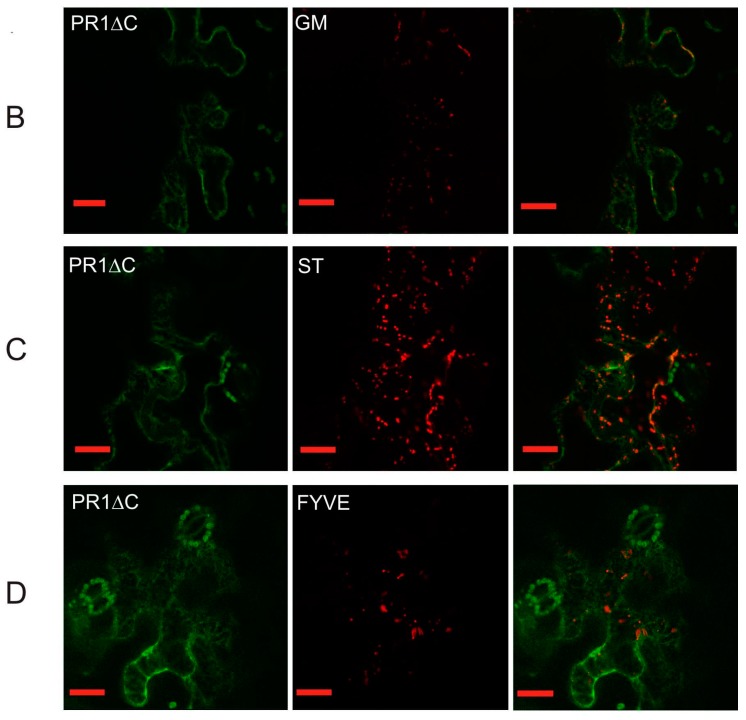
Co-localization of PR1ΔC-GFP with compartmental markers. (**A**) ER marker dsRed-HDEL; (**B**) *cis*-Golgi marker GM-RFP; (**C**) *trans*-Golgi marker ST-RFP; (**D**) PI(3)P-enriched early/late endosomal compartmental marker dsRed-FYVE. Scale bar: 20 μm.

The subset of markers was used for co-localization with the truncated form of PR1, PR1ΔC, which confirmed the previous observation that this version without the sorting signal contained in the C-terminus is localized on the ER (Figure 4).

### 2.3. Localization of AtPR1 Expressed under the Native and 35S Promoter in Arabidopsis Cells

**Figure 5:**
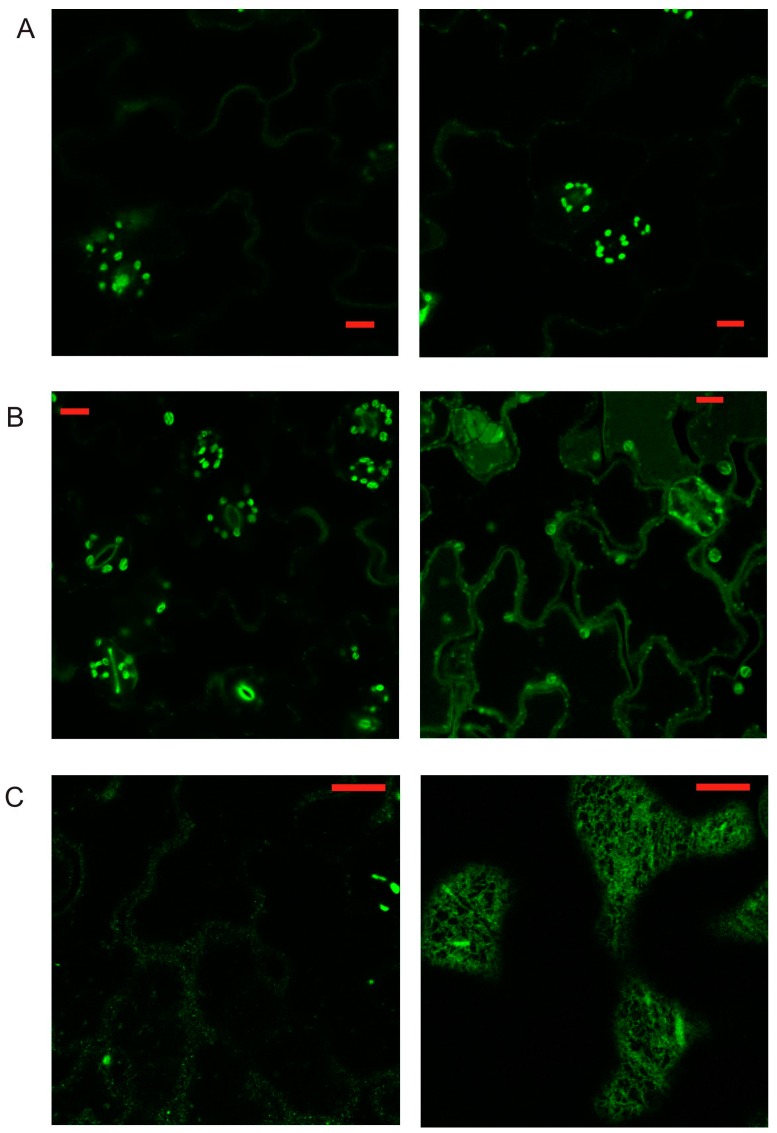
PR1-GFP and PR1ΔC-GFP expressed in *Arabidopsis* under the control of the native promoter after salicylic acid (SA) induction. (**A**) A weak cotyledon signal of PR1-GFP (left) and PR1ΔC-GFP (right) in non-treated seedlings; (**B**) Cotyledon signal for PR1ΔC-GFP (on the right) is slightly stronger than for PR1-GFP (left) for SA-treated seedlings; (**C)** Combination of SA treatment and 24 h dark incubation (the vesicles-like compartment for the full length, and ER localization for truncated PR1 form are fully visible). Scale bar: 10 μm.

**Figure 6:**
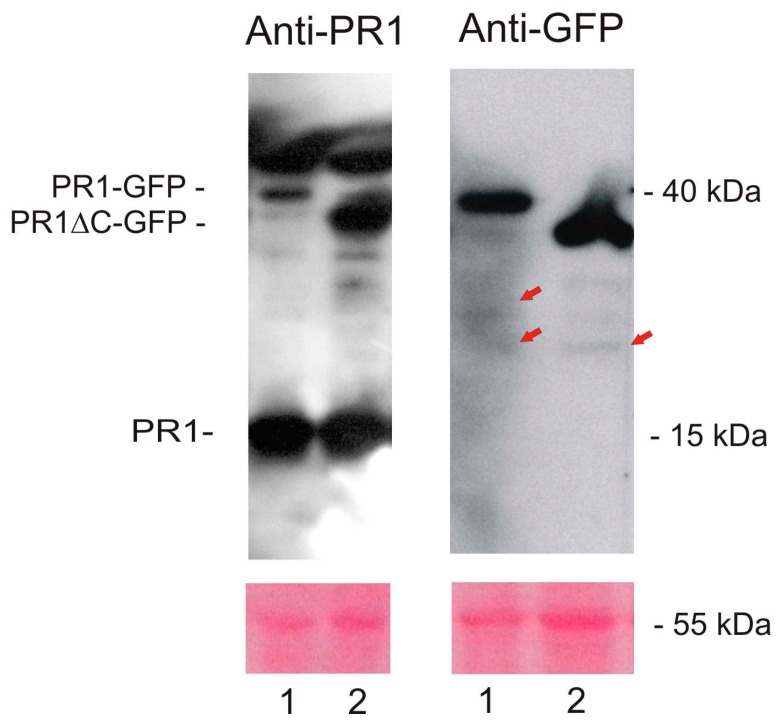
Western blot analysis of total protein extracts from two-week-old SA-treated plants using antibodies raised against PR1 and GFP. 1: An extract from plants carrying promoterPR1::PR1-GFP; 2: An extract from plants carrying prompterPR1::PR1ΔC-GFP. The Ponceau membrane staining of assumed Rubisco band (55 kDa) was used as a loading control. Arrows point to weaker bands that might be products of proteolytical processing of PR1-GFP proteins.

In order to describe PR1 in situ in *Arabidopsis* we have designed PR1-GFP and PR1ΔC-GFP constructs placed under the control of the native 2 kb promoter and used it for the stable transformation of *Arabidopsis*. Without induction, in seven days old seedlings, almost no GFP signal was detected, and after the SA induction of PR1 expression, in cotyledons, we could see only a very weak signal in the case of the full length and slightly stronger for the truncated version of PR1 (Figure 5A,B). In order to check for the proteolysis/degradation of PR1-GFP, we have performed Western blot analysis using antibodies raised against PR1 and GFP (Figure 6). We have detected high amount of endogenous PR1 (15 kDa), and significantly less of GFP tagged constructs (approximately 40 and 35 kDa for the full length and truncated version, respectively). Since the transgenic construct expression is driven by the same promoter, the fact that the expression levels differ indicates that either some distant positive transcriptional signals are missing (out of our 2 kb promoter fragment) in the transgenic PR1s, or the fused protein product is less stable than endogenous PR1. Along some unspecific bands in higher protein mass positions, there were several weaker bands of smaller proteins found with both a PR1-specific antibody that recognizes N-terminal part of PR1, as well as with an anti-GFP body which recognized the intact C-terminal part. These bands might come either from the antibody unspecificity, but also could be signs of the presence of partially proteolytically processed PR1-GFP proteins. In any case, both antibodies recognize well the full length vs. the C-terminally truncated PR1-GFP fusion versions.

Using prolonged (24 h) dark incubation in combination with SA treatment of seedlings, we managed to improve the intensity of GFP constructs signal. This way we could detect the localizations, which were in agreement with previous observations—PR1ΔC-GFP on the ER and ER bodies, and PR1-GFP in a vesicles-like compartment present in the cortical cytoplasm, and also in the vacuole (Figure 5C).

**Figure 7:**
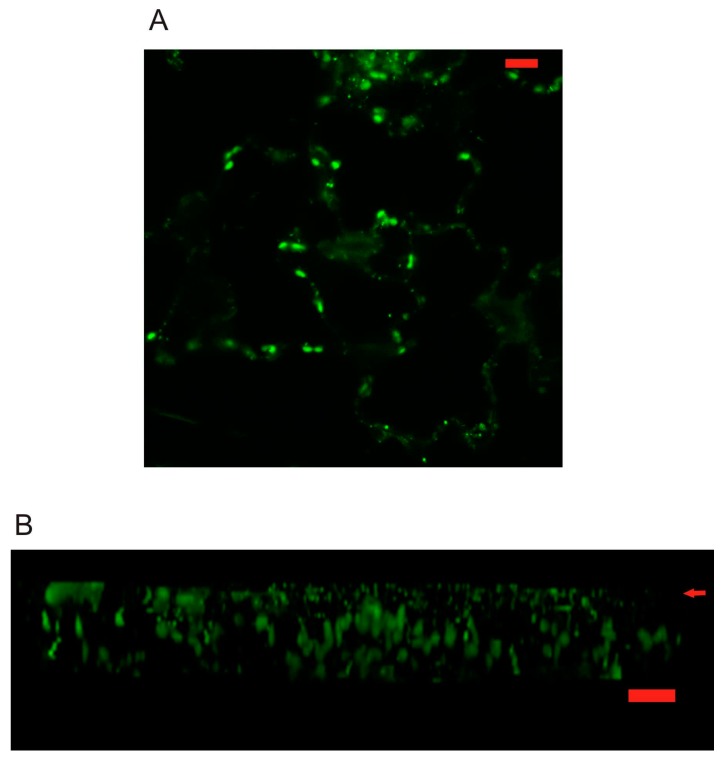
(**A**) *Arabidopsis* overexpression of construct PR1-GFP in cotyledon cells. (**B**) Reconstruction performed for cotyledon epidermal cells allowed the side view/optical cross section which shows that the PR1 vesicles are accumulated just beneath the outer surface (arrow points to outer cortical surface). Scale bar: 10 μm.

In order to further address the identity of PR1-positive intracellular compartment(s), we decided to use stable *Arabidopsis* transformation of PR1-GFP constructs driven by the 35S promoter. This allowed us to obtain a stronger signal without the need to boost the expression by SA, which in high concentrations and prolonged incubations, according to our bright field imaging, significantly affected the appearance and physiology of cells. Under the 35S promoter, in young seedlings, the AtPR1-GFP signal was well detected mainly in ER-bodies like the compartment in cotyledons, probably as an effect of overexpression, but the presence of PR1 compartments similar to those observed in *N. benthamiana* transient expression was also detected (Figure 7A). The three-dimensional (3D) reconstruction performed for cotyledon epidermal cells allowed the side view/optical cross section which showed that the PR1 containing compartments are accumulated just beneath the outer surface (arrowhead, Figure 7B). Bigger bodies observable in inner parts of the cells are autofluorescent plastids.

## 3. Discussion

**Figure 8:**
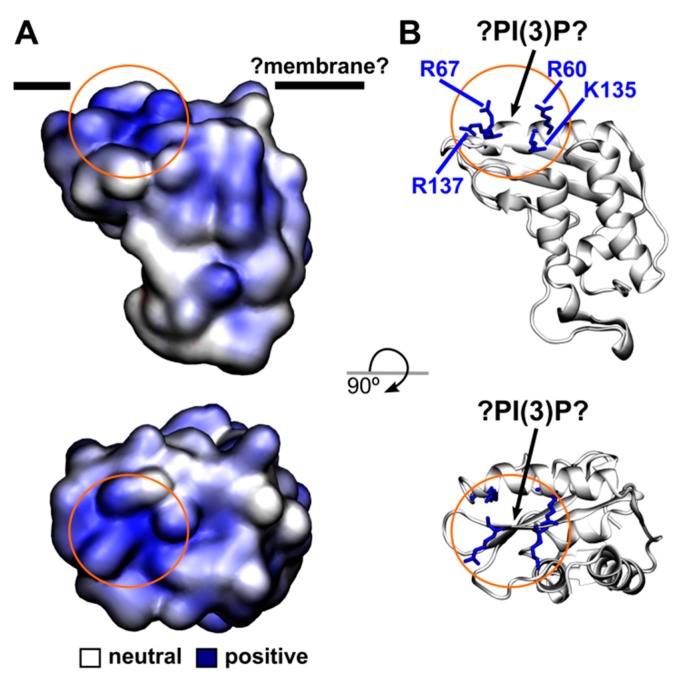
(**A**) Electrostatic potential mapped on the surface of the homology model of the *Arabidopsis* PR1 structure. Black lines represent the position of a hypothetical membrane (?Membrane?) with which PR1 might interact. (**B**) Positively charged amino acid residues (R60, R67, R137, and K135) which could be involved in the interaction with negatively charged phospholipids are shown in blue in the licorice representation. The orange circle highlights a possible position of the PI(3)P molecule.

Here we analyze how the PR1 protein is transported through the cell to the extracellular space. We assayed the localization using the heterologous and transient *Arabidopsis* gene expression in *Nicotiana*. It was known before that in *N. tabacum* there are three acidic (1a, 1b, and 1c) and one basic (1g) PR1 isoforms that are induced upon tobacco mosaic virus (TMV) infection [2]. It is a common knowledge that the basic PR proteins are considered as vacuolar, while acidic as extracellular cargo [22]. However, the situation with PR1s charge and destination may not be that simple, especially in the case of basic AtPR1 that was reported to be secreted [6,12]. In addition, for instance in tomato, homologues of acidic tobacco PR1a, -b, and -c are basic proteins [2]. This shows that individual PR1 orthologs may have different trafficking routes and functions and at this moment, it is not clear how this might be related to their charge. Moreover, from of our results and previously published localization of PR1-RFP [8], it is obvious that one PR1 may have both vacuolar and extracellular localization. In our experiments, a small fraction of PR1 compartments co-localized with Golgi, but the signal overlap with the PI(3)P enriched compartment was clearly dominant. According to Berson et al. [23], Kim et al. [24], and Kolb et al. [25], FYVE marked compartments could be early or late endosomes. Size and morphology of the PR1-associated compartments clearly points to a late endosome identity on the way to maturation to MVBs. The PR1 protein in MVBs could be further targeted to a vacuole, but can be also secreted via exosomes. Targeting to the plasma membrane to release PR1 content into extracellular space, rather than to the vacuole, would be more consistent with the presumed extracellular function of PR1. In our experiments, however, only a weak signal is observed in extracellular space, which can be explained by the fact, that GFP, unlike RFP, is quenched in the acidic cell wall environment. In addition, in stable transformants, after SA induction and prolonged dark treatment, for the full-length construct we see a portion of PR1-compartments in the cortical cytoplasm, and also in intravacuolar bodies. Surprisingly, besides intracellular compartments, we observed for both PR1- and PR1ΔC-GFP association with the plasma membrane, a situation so far unreported for plant PR1s, but well described for the human homolog Golgi-associated PR1 protein (GLIPR2/GAPR1) which was found to interact with the negatively charged membrane phospholipids [26]. We hypothesize that AtPR1 could also directly interact with cellular membranes. To this end, we constructed a homology model of the *Arabidopsis* PR1 protein (see Material and Methods, and Figure 8). Similarly to GLIPR2/GAPR1, the PR1 protein is positively charged and we found one cluster of positively charged amino acid residues (arginines 60, 67, 137 and lysine 135). These amino acid residues could interact with negatively charged phospholipids of the cellular membranes and they would orient the protein towards the lipid bilayer in such a manner that corresponds to the proposed interaction site of GLIPR2/GAPR1 [27]. Moreover, the highly positive surface of *Arabidopsis* PR1 and its localization into the lumen of the MVB, which is surrounded by the FYVE marker, could be explained by a direct interaction of PR1 with PI(3)P. However, this hypothesis needs to be verified by additional experiments, but our data does suggest amino acid residues could be directly involved and in the future tested by site-directed mutagenesis. 

A very interesting and unexpected observation which might be related to the predicted PR1-membrane interaction is a retention of the AtPR1ΔC in the ER when compared to the full length AtPR1. Export or retention from/in the ER are regulated by specific amino acid signals which are best studied in transmembrane secreted proteins (e.g., dilysine motif; [28]); much less is known about soluble or peripheral membrane proteins. It is also possible, that already in this step, the ER membrane enrichment by PI(3)P marks the ER exit sites, similarly to what was found for ER-derived autophagosome formation (PI(3)P is on the cytosolic side of the membrane) [29], or for parasitic *Plasmodium* protein secretion (PI(3)P is on the ER-lumen side) [30]. Interestingly, PR1ΔC lacks several amino acid residues creating the potential PI(3)P-binding site (two β sheets in the predicted PR1 structure are completely missing; Figure S2) and moreover, a model of the mutated protein structure suggests that PR1ΔC is more negatively charged than wild type PR1. The precise topology of PR1–lipid interactions is an important question for future research.

On one side, our PR1 construct is present mainly in a compartment that corresponds to LE/MVB/ prevacuolar endosomal compartment (PVC), on the other side, it also colocalizes with both *cis*- and *trans*-Golgi markers. It remains unsolved whether this ambivalence is an alternative sorting or a successive localization, both possibilities could be also distorted due to overexpressing PR1 conditions which prevented correct protein sorting. We believe that future work using more natural conditions for this protein, such as a pathogen presence, could bring more data on modes of trafficking and secretion of PR1.

## 4. Material and Methods

### 4.1. Plant Material

The seeds of *A. thaliana* Col-0 (Nottingham Arabidopsis Stock Centre, University of Nottingham, UK) were surface-sterilized and plated onto a half-strength Murashige and Skoog medium (1/2 MS). The plants were propagated in vitro for 7 days (23 °C, 16/8 h), and used for treatments and/or microscopical observation, or were transferred into Jiffy tablets and cultivated in a growth chamber under the same cultivation conditions (23 °C, 16/8 h). *N. benthamiana* seeds were surface-sterilized and plated into Jiffy tablets and placed into a growth chamber under the same cultivation conditions (23 °C, 16/8 h).

### 4.2. Cloning Procedure

For preparation and cloning of At2g14610 PR1 constructs, due to the fact that it is an intronless gene, genomic DNA from wild type Col-0 *Arabidopsis* was used as a template for polymerase chain reaction (PCR). Primers enabling cloning into pENTR3C vectors (Thermo Fisher Scientific Inc., Waltham, MA, USA) were EcoORFPR1: 5′-*AAGAATTC*GATGAATTTTACTGGCTATTCTCG-3′ and PR1Xho: 5′-*AACTCGAG*TATGGCTTCTCGTTCACATAATTCC-3′ for the full length open reading frame (ORF) and the same forward but different reverse primer for the C-truncated version PR1ΔXho: 5′-*AACTCGAG*TTCGCAGCGTAGTTGTAGTTAGCC-3′. For cloning of PR1 with and endogenous promoter, the same two reverse primers were used, with EcoPR1: 5′-*AAGAATTC*GATGTTTGAGGTTGAGTACGATGG-3′ as forward. Reverse primers were designed with an omitted STOP codon to allow in-frame fusion with GFP. Cloned constructs were transferred from an entry vector by a recombinant LR clonase of Gateway cloning system (Invitrogen, Thermo Fisher Scientific Inc., Waltham, MA, USA) into pGWB5 (for 35S driven expression) and pGWB4 (for endogenous promoter driven expression) vectors [31]. The constructs were used for the transformation of *Agrobacterium tumefaciens* GV3103 (Nottingham Arabidopsis Stock Centre, University of Nottingham, UK).

### 4.3. Transient Expression in N. benthamiana

Overnight *Agrobacterium* cultures were diluted in infiltration buffer (10 mM MgSO_4_·7H_2_O with 100 μM acetosyringon; both from Sigma (Sigma GmbH, Erlangen, Germany) until optical density (OD) 0.1 and used for infiltration by syringe on the abaxial *N. benthamiana* leaf surface. The fluorescence was observed 48 h later. 

### 4.4. Stable Transformation of A. thaliana

*Arabidopsis* wild type Col-0 was transformed by *Agrobacterium* mediated floral dip method [32]. 

### 4.5. Confocal Microscopy

For live-cell imaging, either infiltrated parts of *N. benthamiana* leaves or 5–7 day-old seedlings were used for observation under the ZeissLSM880 (Carl Zeiss GmbH, Jena, Germany) confocal microscope using ×63 oil immersion objective. Excitation wavelengths used were 488 nm for GFP and 561 nm for RFP and dsRed. The images were analyzed using Zen 2.1 Software (Carl Zeiss GmbH) and Fiji/ImageJ [33,34].

### 4.6. Treatments of Seedlings

In order to boost the expression of PR1 protein from the construct placed under the control of endogenous promoter, SA was used (250–500 μM) for at least 24 h in liquid 1/2 MS in sterile 6-multiwell plates. Seedlings/plants were further used for confocal microscopy or protein extraction. Plasmolysis was induced with 0.8 M mannitol or 1 M NaCl.

### 4.7. Sodium Dodecyl Sulfate–Polyacrylamide Gel Electrophoresis and Western Blot

Total protein extracts were performed from two-week old plants transformed with promoterPR1::PR1-GFP and promoterPR1::PR1ΔC-GFP (after SA induction of PR1 expression) using protein extraction buffer described in Hala et al. [35]. In order to visualize 15 kDa PR1 protein, 15% acrylamide gel was used. Proteins were blotted onto a nitrocellulose membrane (quality of transfer was checked by Ponceau staining) and blocked overnight at 4°C with 5% nonfat dry milk in PBS (137 mM NaCl, 2.7 mM KCl, 10 mM Na_2_HPO_4_, and 2 mM KH_2_PO_4_, pH 7.4) supplemented with 0.2% Tween 20. Primary antibodies anti-PR1 (Agrisera AB, Vanas, Sweeden) and anti-GFP (Roche, Basel, Switzerland) were diluted 1:1000 and incubated with the membranes for 3 h at room temperature in the blocking solution. Horseradish peroxidase-conjugated antibody (Promega, Madison, WI, USA) was applied followed by enhanced chemiluminescent ECL detection (Amersham, GE Healthcare, Chicago, IL, USA).

### 4.8. Homology Modelling of the PR1 Structure and the Electrostatics Calculation

The homology model of *Arabidopsis* PR1 and PR1ΔC was built using the SWISS-MODEL portal [36] and evaluated by the ProSa-Web server [37]. Electrostatic potential of the protein was calculated by numerically solving the nonlinear Poisson–Boltzmann equation in the APBS program [38]. The VMD program [39] was used to prepare Figure 8 and Figure S2.

## Supplementary Materials

Supplementary materials can be found at www.mdpi.com/1422-0067/18/4/825/s1.
