# Supplementary material for: Subcellular Localization of Arabidopsis Pathogenesis-Related 1 (PR1) Protein
Source: Int J Mol Sci. 2017 Apr 13;18(4):825. doi: 10.3390/ijms18040825 (PMC5412409; doi:10.3390/ijms18040825)
Supplement: Supplementary file 1 [file ijms-18-00825-s001.zip › Subcellular Localization of Arabidopsis Pathogenesis‐Related 1 (PR1) Protein.pdf]

Supplementary Materials: Subcellular Localization of *Arabidopsis* Pathogenesis-Related 1 (PR1) Protein

Tamara Pečenková, Roman Pleskot and Viktor Žárský

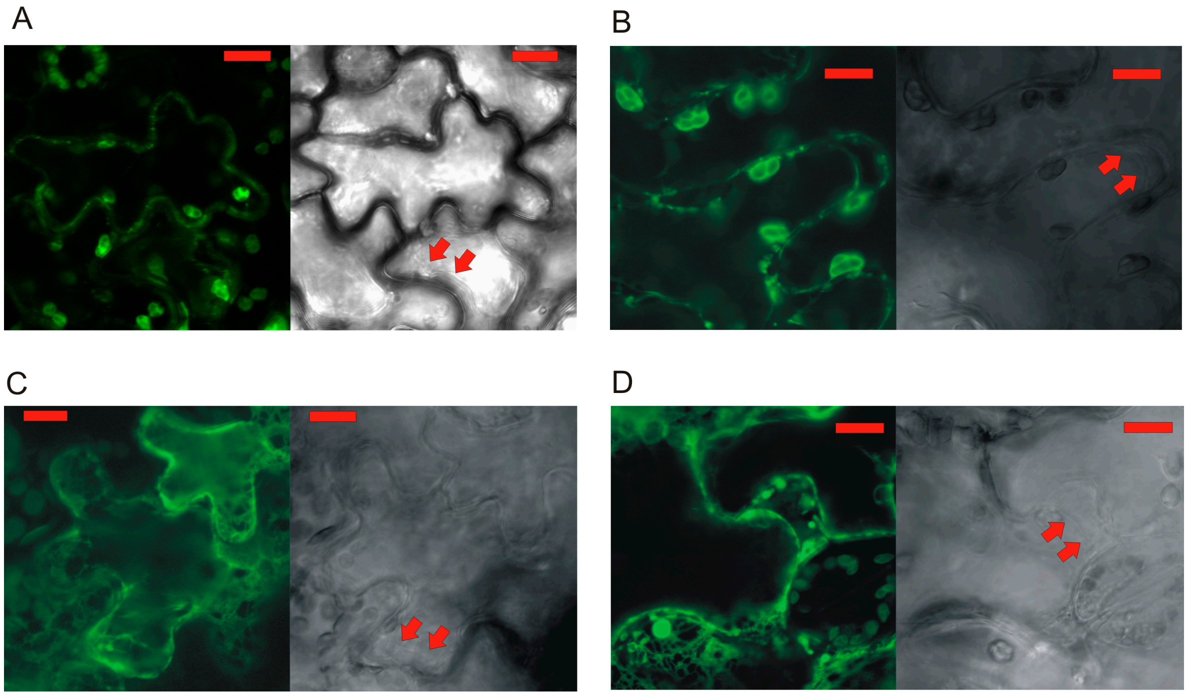

Figure S1. Mannitol and NaCl induced plasmolysis of leaf cells of *N. benthamiana* with transiently expressed PR1-GFP and PR1ΔC-GFP. Mannitol (**A**) and NaCl (**C**) induced plasmolysis of leaf cells of *N. benthamiana* with transiently expressed PR1-GFP; PR1ΔC-GFP in mannitol (**B**) and NaCl (**D**) induced plasmolysis. In both cases, the observation was performed in phosphate buffer with pH 9. Arrows point to spaces between the protoplast outlines and cell wall. Scale bar: 10 µm.

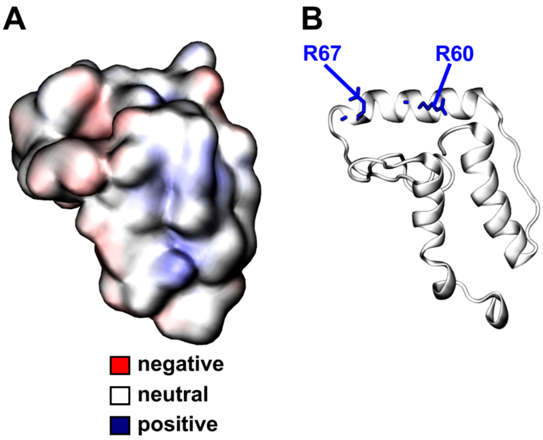

Figure S2. Electrostatic potential mapped on the surface of the homology model of the *Arabidopsis* PR1ΔC structure. (**A**) Electrostatic potential mapped on the surface of the homology model of the *Arabidopsis* PR1ΔC structure. (**B**) PR1ΔC lacks several positively charged amino acid residues which could be involved in the interaction with negatively charged phospholipids.

Video S1. Time-series scanning of PR1-GFP-positive vesicles. The strong signal of big body in the cytoplasm is autofluorescence of chloroplast. Scale bar: 10 µm.

Video S2. Time-series scanning of PI(3)P-PR1-GFP-positive vesicles.
